# Using a Novel, Subconjunctival, Sustained-Release Mitomycin C Formulation in a Rabbit Model of Filtration Surgery with Gel Stent Implantation

**DOI:** 10.1089/jop.2023.0100

**Published:** 2024-06-17

**Authors:** Susan S. Lee, Saumya Nagar, Lakshmi Rajagopalan, Werhner Orilla, Karl G. Csaky, Alexandra Almazan, Liuqing Yang, Michael R. Robinson

**Affiliations:** ^1^ Allergan, an AbbVie company, Irvine, California, USA.; ^2^ Retina Foundation of the Southwest, Dallas, Texas, USA.

**Keywords:** drug delivery, gel stent, minimally invasive glaucoma surgery, mitomycin C, rabbit model, subconjunctival filtration surgery, XEN

## Abstract

**Purpose:**

To investigate gel stent implantation with and without intraoperative sustained-release mitomycin C (MMC SR) in a rabbit model for gel stent implantation, and to examine aqueous humor outflow (AHO) postimplantation.

**Methods:**

Four groups of rabbits were included. Group 1 was untreated (control). Groups 2, 3, and 4 received the gel stent without MMC, with MMC solution (subconjunctival injection), and with MMC SR (subconjunctival injection), respectively. Intraocular pressure (IOP) and AHO were assessed via tonometry and indocyanine green-based angiography, respectively. The main efficacy measure was change in IOP from baseline.

**Results:**

Following gel stent implantation, Groups 2, 3, and 4 maintained ≥20% IOP reduction (response) for a median duration of 1 week, 6.5 weeks, and 30 weeks, respectively. Angiography showed normal aqueous humor drainage (Group 1) beginning at the perilimbal trabecular plexus and continuing posteriorly to episcleral outflow vessels. Following implantation, drainage occurred preferentially and directly into the subconjunctival bleb.

**Conclusions:**

Gel stent implantation with MMC SR was most effective in achieving sustained, long-term IOP reduction in the rabbit model, compared with implantation with or without MMC solution. Bleb presence and the postimplantation aqueous angiography results indicated redirection of the AHO to the subconjunctival vasculature and presumed lymphatics, suggesting efficient glaucoma filtration to lower IOP in this model. This rabbit model and aqueous angiography may help refine understanding of the mechanism of action of minimally invasive glaucoma surgeries and ultimately translate to improved surgical devices and procedures for patients with glaucoma.

## Introduction

Glaucoma is predicted to affect 111.8 million individuals worldwide by 2040.^1^ It is characterized by the degeneration of retinal ganglion cells, which is related to the intraocular pressure (IOP) and underlying balance between aqueous humor production and outflow.^2–4^ In humans, in the conventional aqueous humor outflow (AHO) pathway, aqueous humor flows from the anterior chamber through the trabecular meshwork and Schlemm's canal to the collector channels, the intrascleral venous plexus, and then the aqueous and episcleral veins.^5–7^ In the unconventional AHO pathway, aqueous humor flows from the anterior chamber to the ciliary body, to the suprachoroidal space, and then exits via the vasculature/sclera.^8,9^

The objective of current glaucoma surgical treatments is to improve AHO and reduce IOP. Glaucoma surgeries range from incisional procedures, such as trabeculectomy, to minimally invasive glaucoma surgery (MIGS) and minimally invasive bleb-forming surgery (MIBS) devices that bypass ocular structures of high resistance to drain aqueous humor to areas of lower resistance.^10^ The XEN^®^45 Glaucoma Treatment System (gel stent; Allergan, an AbbVie company, Irvine, CA) is a MIBS device that facilitates AHO from the anterior chamber to the subconjunctival space.^11^ It has been shown to lower IOP for at least 3 years in humans^12–16^ and to be noninferior to trabeculectomy at 1 year based on the proportion of patients achieving ≥20% IOP reduction from baseline without increase in IOP-lowering medication, clinical hypotony, vision loss to counting fingers, or secondary surgical intervention.^17^

One challenge of all glaucoma filtration surgeries is postoperative fibrosis/scarring of the filtration path, which ultimately leads to AHO blockage and IOP increase. Antifibrotics/antimetabolites, such as mitomycin C (MMC), are thus frequently used to prevent subconjunctival tissue scarring and ensure long-term IOP lowering following glaucoma filtration surgeries,^18–23^ including gel stent implantation.^11,24,25^ MMC, however, must be used cautiously to avoid potentially serious complications.^26^ A slow-released formulation of MMC would thus be helpful to control the scarring process and reduce fibrosis over time, while reducing the dose of MMC to minimize toxicity.

Unlike human eyes, rabbit eyes do not have a well-defined Schlemm's canal, but have an analogous aqueous plexus instead. In the conventional rabbit AHO pathway, aqueous humor thus flows through the angular meshwork, angular aqueous plexus, collector channels, and then aqueous veins.^27,28^ Despite this difference, rabbits have been/are being used to investigate scar formation and various pharmacologic interventions to reduce postoperative fibrosis and bleb failures after incisional filtration surgery.^29–36^ To the best of our knowledge, however, only one study has been published to date, in which a rabbit model is being developed to investigate methods of preventing postoperative fibrosis and bleb failure following ab-interno placement of a novel MIGS device.^34^

In this study, we investigated ab-interno implantation of the gel stent with and without intraoperative MMC (solution vs. sustained release [SR] formulation) in a rabbit model for MIBS, and examined IOP reduction and bleb survival. In addition, aqueous angiography was used to assess AHO before and after gel stent implantation.

## Materials and Methods

### Animals

This study complied with all requirements and regulations issued by the United States Department of Agriculture regarding the Animal Welfare Act, 9 CFR, Parts 1, 2, and 3. The protocol was approved by Allergan's Animal Care and Use Committee (AACUC), and all procedures were performed in accordance with the Association for Research in Vision and Ophthalmology (ARVO) Statement for Use of Animals in Ophthalmic and Vision Research.

Adult female Dutch Belted (DB) and New Zealand White (NZW) rabbits (6–10 months, 2–4 kg) were used as follows: Group 1, untreated control (DB, *n* = 4); Group 2, gel stent implantation without MMC (NZW, *n* = 7); Group 3, gel stent implantation with MMC solution (NZW, *n* = 9); and Group 4, gel stent implantation with MMC SR (NZW, *n* = 4). Group 3 was the largest group because it was used in more experiments than the other groups.

Since Group 2 was expected to exhibit bleb fibrosis and failure more rapidly than Groups 3 and 4, the number of animals in this group was also increased (*N* = 7) to ensure that the IOP-related outcomes (defined in “Outcome measures and statistical analysis”) could be assessed. Otherwise, females were used as they are less aggressive than their male counterpart, making handling safer. Animals were housed individually in mesh cages, maintained on a 12–14-h light/dark cycle, and routinely monitored for food consumption and signs of pain or distress.

After initial AHO assessments were performed in DB rabbits, angle-related findings indicated that the eyes of NZW rabbits were better suited for gel stent implantation, due to their larger angle size, hence the switch to NZW rabbits for Groups 2–4.

### Angle assessment

**FIG. 1.**
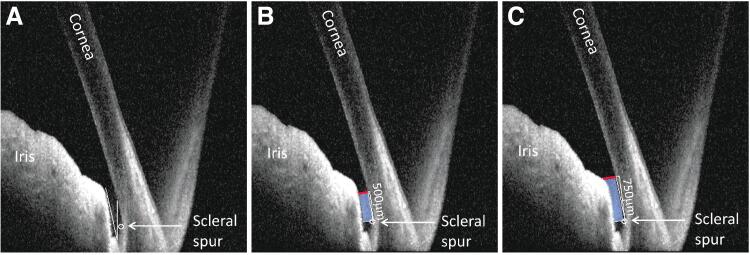
Representative OCT images showing the **(A)** anterior chamber angle, **(B)** AOD 500 (*red line*) and TISA 500 (*blue shaded area*), and **(C)** AOD 750 (*red line*) and TISA 750 (*blue shaded area*) in the rabbit model. The numbers 500 and 750 refer to the distance from the scleral spur at which the perpendicular line was drawn. AOD, angle opening distance; OCT, optical coherence tomography; TISA, trabecular-iris space area.

Before gel stent implantation could be performed, iridocorneal angles were assessed bilaterally in the superotemporal quadrant of 4 DB and 6 NZW rabbits, using anterior segment optical coherence tomography (AS-OCT) imaging (Spectralis OCT; Heidelberg Engineering, Germany). Angle parameters, determined using ImageJ software (National Institutes of Health, Bethesda, MD),^37,38^ included the following: angle opening distance (AOD; distance [mm] along the line drawn perpendicularly from the end of Descemet's membrane to the iris surface); anterior chamber angle (ACA; angle between the iris tangential line [at the first iris bulge] and the posterior corneal surface tangential line, with its apex in the angle recess); and trabecular-iris space area (TISA; polygonal area [mm^2^] formed by the anterior AOD; a line drawn from the scleral spur perpendicular to the plane of the inner scleral wall to the iris; the corneoscleral wall; and the inferior iris surface) (Fig. 1).

AOD 500 and TISA 500 were calculated based on the perpendicular line drawn 500 μm from the scleral spur (Fig. 1). AOD 750 and TISA 750 were calculated based on the perpendicular line drawn 750 μm from the scleral spur (Fig. 1).

### Evaluation of AHO via aqueous angiography and OCT

Imaging was to be performed in DB rabbits (Group 1, *n* = 4) and NZW rabbits (Group 3, *n* = 5) before and after implantation (described in “Ab-interno placement of the gel stent”). Following application of a topical anesthetic (proparacaine or tetracaine) to the eye, topical betadine 5% (antiseptic) was applied to the eye and surrounding fur for 2 min and washed out with balanced salt solution (BSS). Animals were then anesthetized via intravenous administration of ketamine (7.5–10 mg/kg) and xylazine (1–10 mg/kg), after which the eye was rinsed with betadine 5% before receiving 1‒2 drops of 0.5% proparacaine HCl.

Following intracameral injection of 5 μL of a 2.5 mg/mL solution of indocyanine green (ICG; Akorn Pharmaceutical, Lake Forest, IL), the AHO vasculature was serially imaged/captured in videos (30–60 s each) at 10-min intervals for up to 60 min, using the confocal scanning laser ophthalmoscopic feature of the Spectralis Heidelberg Retina Angiograph (HRA)+OCT platform that allows angiography and OCT images to be acquired simultaneously.

### Ab-interno placement of the gel stent

NZW rabbits (Groups 2–4) underwent implantation of the gel stent (inner diameter, 45 μm) in the left eye using a modified ab-interno approach. To accommodate the rabbit anatomy and facilitate gel stent implantation, the gel stent was transferred from its original applicator to a sterile applicator similar in design to that of the bimatoprost implant (Durysta; Allergan, an AbbVie company),^39^ which has a smaller needle (28 G thin-wall needle; TSK Laboratory, Vancouver, British Columbia, Canada) than the gel stent applicator (27 G).

To standardize implantation across all animals, a small subconjunctival bleb was also raised in the superotemporal quadrant by injecting 20 to 50 μL of BSS with a 30 G needle on a 1-mL syringe. The conjunctiva was grasped at the limbus in the superotemporal quadrant, and the eye was reflected down. The 28 G needle of the customized applicator (preloaded with the gel stent) was advanced through the cornea superotemporally (2–3 mm from the limbus) and the iridocorneal angle. The 28 G needle was advanced a total of 4–5 mm, with the bevel exiting 1 mm posterior to the limbus into the subconjunctival space. Injection through the needle proceeded, leaving 1–2 mm of the 6-mm gel stent in the anterior chamber. Positioning of the gel stent was adjusted as/if needed with forceps (from the conjunctival side), and the gel stent expanded and became flexible upon hydration (Supplementary Video S1).

Immediately after implantation, bleb formation/presence was visually confirmed to ensure proper placement and function of the gel stent. Groups 3 and 4 received a subconjunctival injection of MMC solution (Leiters, Englewood, CO; 50 μL of 0.2-mg/mL solution; 10 μg total) or MMC SR (a proprietary sustained-release injectable gel formulation in development), respectively, at the implantation site, toward the distal end of the implant. Group 2 did not receive MMC. A single subcutaneous injection of buprenorphine 0.04 mg/kg was then administered (Groups 2–4), and the eye was flushed topically with sterile saline and treated with 1–2 drops of antibiotic (Zymaxid [0.5% gatifloxacin ophthalmic solution; Allergan, an AbbVie company]) for at least 3 consecutive days postimplantation.

Postoperative care included ocular examinations performed daily during week 1 postimplantation, and then once a week for evidence of inflammation, discharge, squinting, ocular discomfort, or other safety/tolerability issues. To monitor placement of the gel stent in the iridocorneal angle and subconjunctival space, anterior segment photographs and AS-OCT images of the filtration site were obtained at weekly intervals for the first 4 weeks, and as deemed warranted thereafter.

### Monitoring of filtration blebs over time

Subconjunctival blebs were categorized as visually present or absent by visual inspection and anterior segment photographs obtained daily for the first week and as deemed warranted thereafter, in all eyes implanted with the gel stent (Groups 2–4).

### IOP measurements

For each animal of each group, IOP was measured bilaterally (using the Model 30™ Pneumatonometer; Reichert, Inc., Depew, NY) on day −1 (before implantation), as well as day 2 and weeks 1, 2, 4, 6, 8, 10, 12, 16, 18, 20, 22, 24, and 30 postimplantation, as long as there was evidence of IOP lowering. If there was no IOP lowering on a given week, no measurements were taken on subsequent weeks.

IOP was assessed at approximately the same time/hour on each evaluation day, before the angiography and OCT imaging, and by the same evaluator. Each assessment consisted of 2 consecutive measurements in each eye, while the animal was awake and restrained; if the difference between them was ≤2 mmHg, the IOP value for the given eye was the mean of both measurements. Otherwise, a third measurement was taken, and the IOP value for the given eye was the median of all 3 measurements. Data were expressed as mean (standard error of the mean [SEM]) percentage IOP reduction from baseline.

### Outcome measures and statistical analysis

The main outcome measure of efficacy was IOP. Responders were defined as having at least 20% IOP reduction from baseline during the first week following gel stent implantation. A Kaplan–Meier analysis was performed among responders with a log-rank test to evaluate the probability of maintaining ≥20% IOP reduction from baseline. A measurement of less than 20% IOP reduction from baseline constituted an event. The duration of response was then evaluated in all responders. The association between IOP reduction persistence and MMC treatment (solution vs. SR) was also evaluated using a repeated measures mixed effects model with random intercept to account for within-animal correlation. The between-group mean differences were tested in a pair-wise manner using Fisher's least significant difference test.

Parameters from the eye with a better (larger) iridocorneal angle were analyzed using an unpaired one-tail *t* test. All analyses were performed with GraphPad Prism Version 9 (GraphPad Software, San Diego, CA). A *P* value <0.05 was considered statistically significant.

Safety of the gel stent implantation procedure was assessed as the incidence of surgical complications and postoperative adverse events, and presented as frequency counts and percentages. OCT was also used to check for proper placement of the gel stent in the iridocorneal angle.

## Results

### Angle assessment

Before any glaucoma surgery can be performed in patients, assessment of their iridocorneal angle is essential to determine whether there is enough room to accommodate a given implant or device.^40^ Similarly, implantation of the gel stent in rabbits required prior confirmation that their angle was large enough to accommodate the implant (to avoid injury to adjacent structures). The AOD 500 and AOD 750 values (SEM) were 0.185 (0.016) mm and 0.239 (0.044) mm in NZW rabbits, and 0.155 (0.035) mm and 0.205 (0.034) mm in DB rabbits, respectively. The TISA 500, TISA 750, and ACA values (SEM) were 0.089 (0.007) mm^2^, 0.137 (0.012) mm^2^, and 10.697 (1.119) in NZW rabbits, and 0.059 (0.020) mm^2^, 0.106 (0.030) mm^2^, and 6.446 (1.687) in DB rabbits, respectively.

**FIG. 2.**
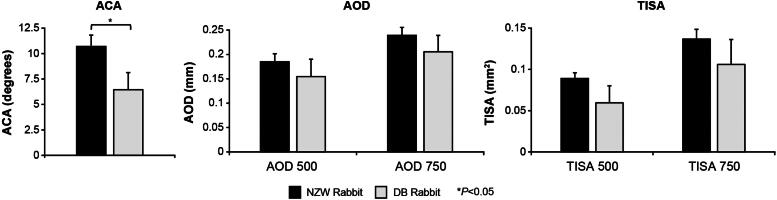
Comparisons of ACA, AOD, and TISA between NWZ (*n* = 6) and DB (*n* = 4) rabbits. Per Fig. 1, 500 and 750 refer to the distance from the scleral spur at which the perpendicular line was drawn. ACA, anterior chamber angle; DB, Dutch Belted; NZW, New Zealand White.

There was a general trend toward larger angle parameters overall in NZW rabbits than DB rabbits (Fig. 2), and the ACA was statistically significantly larger in NZW rabbits than DB rabbits (*P* < 0.05; Fig. 2).

### Effects of gel stent implantation on AHO

**FIG. 3.**
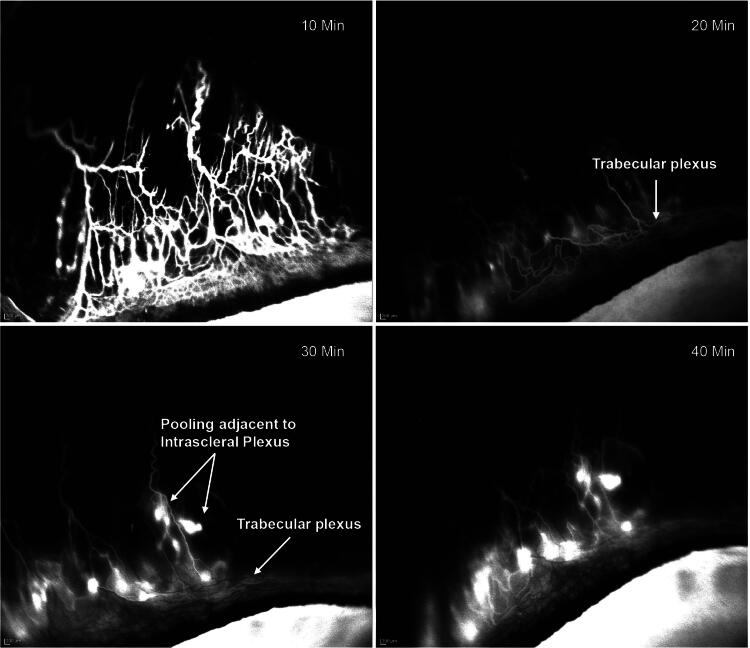
Angiography-based visualization of the dynamic, real-time aqueous humor outflow following ICG injection in the anterior chamber of a control rabbit representative of Group 1 (control, *n* = 4) showed rapid filling of outflow vessels with ICG. Over time, localized pooling/accumulation of ICG was observed adjacent to the intrascleral plexus while the trabecular plexus signal gradually reduced. Images in Figs. 3–6 were obtained with the same angiography protocol. ICG, indocyanine green; min, minutes.

In untreated/control rabbits (Group 1), AHO showed segmental angiographic patterns beginning at the perilimbal trabecular plexus as early as 10 min post-ICG injection (Fig. 3). The aqueous humor drained posteriorly into the radially oriented episcleral outflow vessels, which are heavily concentrated at the 12 o'clock position, over the superior rectus muscle. The dynamic nature of AHO was observed as vessels rapidly filled with ICG and, over time, small areas of ICG pooling/accumulation were observed adjacent to the intrascleral plexus, while the trabecular plexus signal intensity gradually decreased (Fig. 3).

**FIG. 4.**
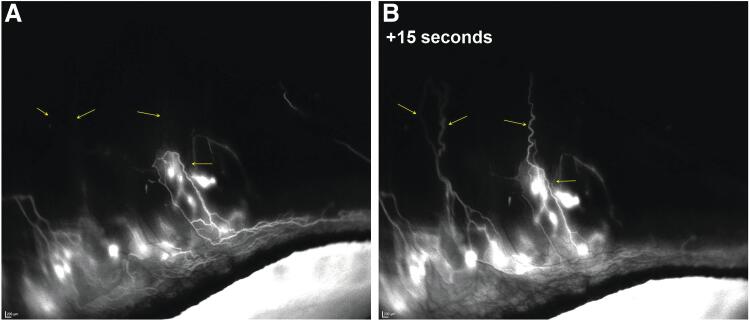
Pulsatile aqueous humor outflow in a control rabbit representative of Group 1 (*n* = 4) is visualized as dark episcleral vessels **(A)** that filled with aqueous humor (ICG) 15 s later **(B)**. The magnification, image settings (eg, background brightness/intensity), and recording parameters were the same in **(A, B)**, and the *arrows* highlight the differences between panels. Images in Figs. 3–6 were obtained with the same angiography protocol.

AHO also appeared to drain into deep, radially oriented, large-diameter episcleral and intrascleral vessels, and real-time imaging showed that the aqueous humor drainage was pulsatile in these episcleral and intrascleral vessels (Fig. 4). Notably, fellow control eyes exhibited no ICG signal, confirming that the signal detected in the eyes injected with ICG was due to aqueous drainage, as opposed to ICG in the systemic circulation. These findings (Figs. 3 and 4) are relevant as they show that the rabbit model reproduces AHO features observed in humans and thus support use of rabbits as a model to study gel stent implantation and postoperative fibrosis, despite the anatomical differences mentioned in the Introduction.

**FIG. 5.**
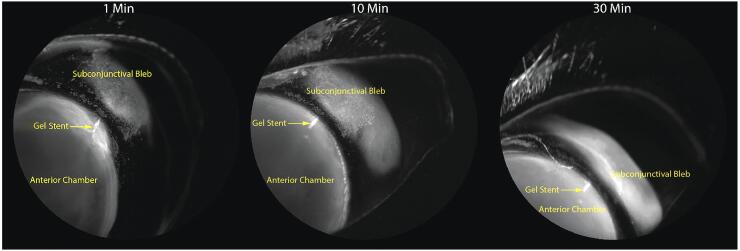
Dynamic, real-time aqueous humor angiography images (appropriately calibrated) in a NZW rabbit implanted with the gel stent and MMC solution, representative of Group 3 (*n* = 5), shows that the ICG dye diffuses out toward the boundaries of the bleb, due to preferential drainage of the aqueous humor through the gel stent that provides the route of least resistance. Images shown in Figs. 3–6 were obtained with the same angiography protocol. MMC, mitomycin C.

**FIG. 6.**
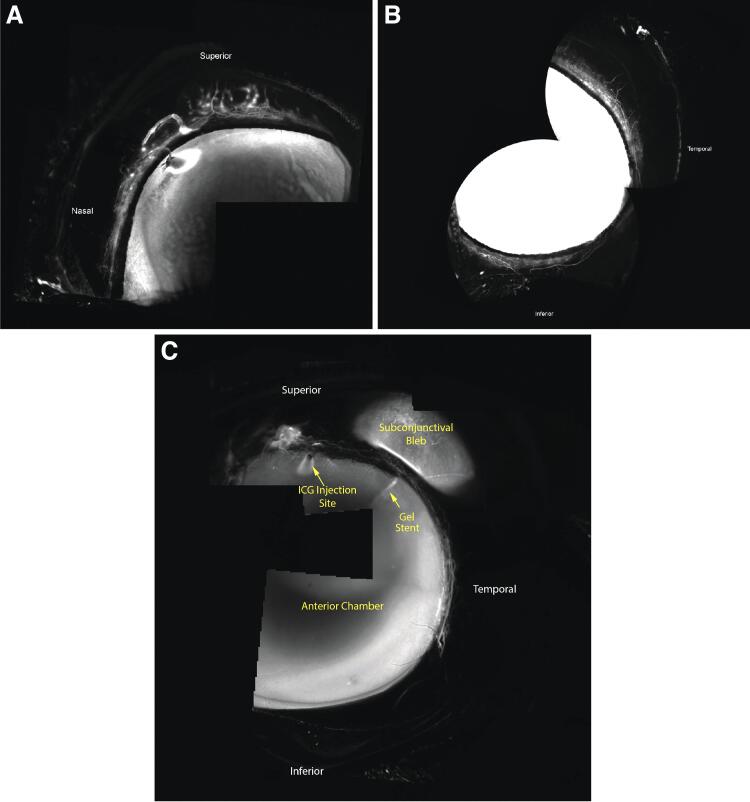
AHO before and after gel stent implantation with MMC solution in a NZW rabbit representative of Group 3 (*n* = 5). **(A)** Normal AHO before gel stent implantation, visualized with the ICG dye, shows that drainage occurs through vessels of the perilimbal trabecular plexus in the nasal and superior quadrants. **(B)** Additional vessels of the perilimbal trabecular plexus from the inferior and temporal quadrants are also used for drainage in normal AHO. **(C)** Following gel stent implantation, AHO preferentially flows through the stent and fewer regional vessels are positive for AHO/ICG. Images shown in Figs. 3–6 were obtained with the same angiography protocol. AHO, aqueous humor outflow.

Groups 2 and 3 were imaged immediately following gel stent implantation and showed preferential drainage directly into the subconjunctival bleb as early as 30 s post-ICG injection. Over a short time, the ICG gradually mixed with the aqueous humor within the bleb and diffused toward the bleb boundaries (Fig. 5). Unlike normal AHO in the conventional outflow pathway, which occurs in all quadrants through the perilimbal trabecular plexus of outflow vessels (Fig. 6A, B), less angiographic signal was seen through the plexus following gel stent implantation, indicating efficient and preferential drainage of aqueous humor through the gel stent to the subconjunctival bleb (Fig. 6C).

The prominent segmental conventional outflow observed in untreated/control rabbits was indeed less apparent in rabbits that received the gel stent, as radially oriented outflow vessels were not observed superiorly and inferiorly (Fig. 6C). This change, upon gel stent implantation, shows that aqueous humor angiographic patterns have the potential to be dynamic.

### OCT imaging of AHO

**FIG. 7.**
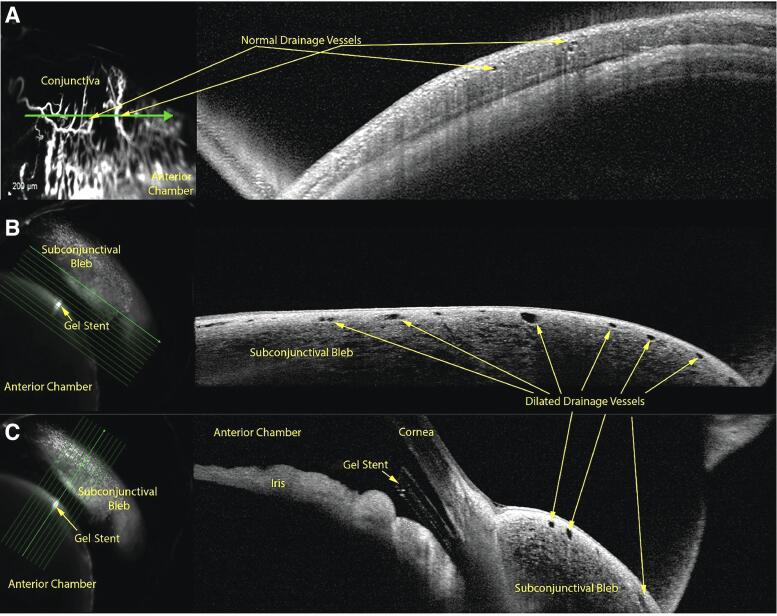
Optical coherence tomography cross-sectional images of outflow vessels acquired simultaneously with angiography in a NZW rabbit representative of Group 3 (*n* = 5) **(A)** before and **(B, C)** after gel stent implantation with MMC solution.

In Group 1 (untreated), the aqueous humor drained posteriorly through conjunctival episcleral or intrascleral vessels, as shown on OCT cross-sectional images of the vessel lumens acquired simultaneously with angiography (Fig. 7A). In Group 3, OCT imaging of the blebs following gel stent implantation showed a greater number of superficial conjunctival lumens that were also enlarged (Fig. 7B, C), consistent with enhanced aqueous humor drainage through the bleb and reminiscent of lymphatic outflow lumens, compared with the normal drainage pathways. Visually speaking, the subconjunctival blebs appeared to be uniform and diffuse, which likely contributed to the efficient aqueous humor drainage in implanted rabbits.

### Bleb presence

**FIG. 8.**
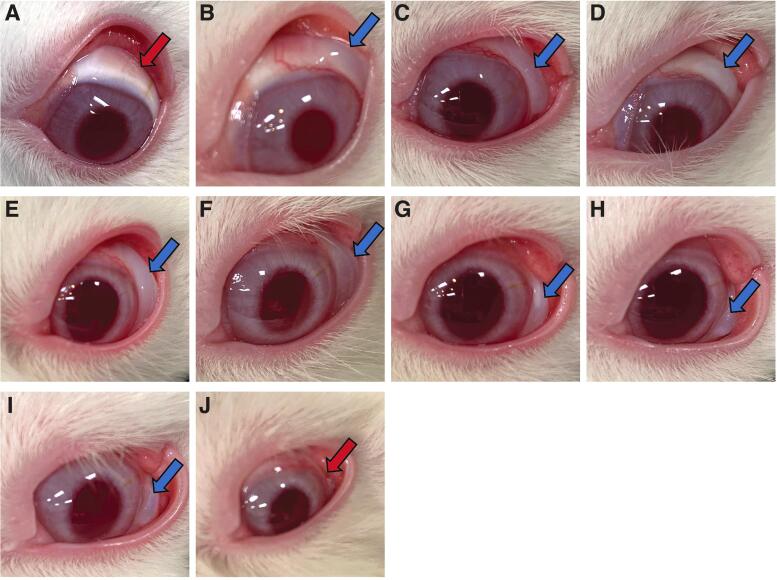
Bleb presence/persistence in NZW rabbits following gel stent implantation. **(A)** The image is from a representative rabbit in Group 2 (no MMC, *n* = 7) at 2 weeks postimplantation. **(B–D)** The images are from one representative rabbit in Group 3 (MMC solution, *n* = 9) at 2 **(B)**, 4 **(C)**, and 11 **(D)** weeks postimplantation. **(E–J)** The images are from one representative rabbit in Group 4 (MMC SR, *n* = 4) at 2 **(E)**, 4 **(F)**, 11 **(G)**, 22 **(H)**, 24 **(I)**, and 30 **(J)** weeks postimplantation. The *red arrows* point to the absence of a bleb. The *blue arrows* point to the presence of a bleb. SR, sustained release.

Immediately after gel stent implantation, blebs were visually present in all rabbits (Groups 2–4). In Group 2, the blebs were absent by week 2 postimplantation (Fig. 8A). In contrast, blebs persisted and were still visually present at week 11 in Group 3 (Fig. 8B–D) and week 24 in Group 4 (Fig. 8E–J).

### IOP reduction from baseline following gel stent implantation

**FIG. 9.**
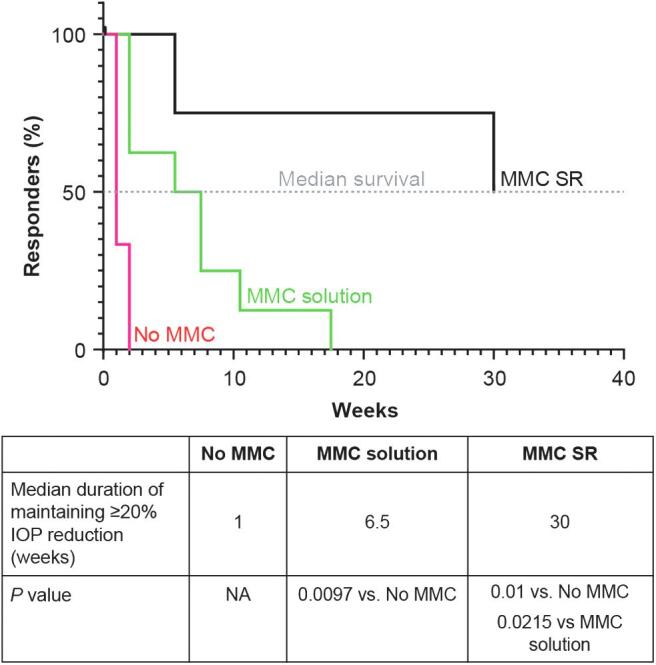
Percentage of NZW rabbits exhibiting ≥20% IOP reduction from baseline (responders) over time after the gel stent implantation without MMC (Group 2, *n* = 4), with MMC solution (Group 3, *n* = 8), and with MMC SR (Group 4, *n* = 4). The median survival time was the time (in weeks) at which half of the rabbits were still exhibiting ≥20% IOP reduction from baseline following gel stent implantation without MMC (*red curve*), with MMC solution (*green curve*), and with MMC SR (*black curve*). IOP, intraocular pressure; NA, not applicable.

For this analysis, only animals categorized as responders during the first week postimplantation were studied and used to determine statistical significance of the findings, that is, 4 of 7 in Group 2, 8 of 9 in Group 3, and 4 of 4 in Group 4. In Group 2 (implantation without MMC), no rabbits/eyes exhibited ≥20% IOP reduction from baseline by week 2. In Group 3 (implantation with MMC solution), no responder eyes were exhibiting ≥20% IOP reduction at week 17.5. In Group 4 (implantation with MMC SR), however, half of the responder eyes were still exhibiting ≥20% IOP reduction at week 30 (Fig. 9). The median survival times of 1 week (Group 2), 6.5 weeks (Group 3), and 30 weeks (Group 4) indicated that gel stent implantation with MMC SR sustained IOP reduction over a statistically significantly longer period of time, compared with implantation with or without MMC solution (*P* ≤ 0.0215; Fig. 9).

**FIG. 10.**
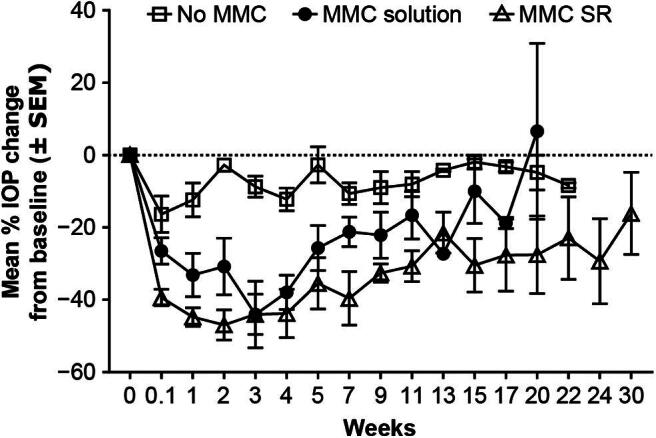
Mean IOP change over time in NZW rabbits that underwent gel stent implantation without MMC (Group 2, *n* = 7), with MMC solution (Group 3, *n* = 9), and with MMC SR (Group 4, *n* = 4). SEM, standard error of the mean.

Consistent with the survival curves, mean IOP reductions of 2.8% (Group 2), 30.8% (Group 3), and 46.9% (Group 4) were recorded at week 2. At week 20, Group 4 was still exhibiting 27.6% IOP reduction from baseline, compared with low or no IOP reductions in Groups 2 and 3 (Fig. 10). Notably, in calculations of the between-treatment difference in predicted means over 20 weeks postimplantation (latest time point common to Groups 2–4), a statistically significantly greater IOP reduction from baseline was detected across all time points with MMC SR (≥14.3%), compared with MMC solution or no MMC (*P* ≤ 0.0344).

### Safety

The gel stent was well tolerated. No significant surgical or bleb-related complications, postoperative infections, unexpected animal deaths, inflammation requiring topical or systemic corticosteroids, or other adverse events occurred at any time during the study. OCT images showed proper gel stent placement in the iridocorneal angle, without postoperative trauma to the corneal endothelium or iris. Notably, there were no cases of persistent hypotony. Similarly, no cases of transient hypotony were observed, which could be due to the postoperative IOP assessment schedule starting on day 2.

## Discussion

Our results showed that gel stent implantation (ab-interno) with or without a single intraoperative, subconjunctival injection of MMC solution or MMC SR was well tolerated in rabbits, and that MMC SR was most effective in achieving sustained IOP reduction from baseline. Compared with MMC solution, MMC SR favored formation of filtration blebs that persisted longer, consistent with a longer duration of IOP lowering and greater responder rate over time.

While various animal studies have investigated ways to minimize subconjunctival scarring, extend bleb survival, and achieve lasting IOP reduction following glaucoma surgery,^35,41–47^ few involved MIGS or MIBS^34,36,41,42^ and none (published to date) involved ab-interno procedures performed with and without an antifibrotic. Because the eyes of mice and rats are not large enough to accommodate the 6-mm-long gel stent,^48^ and monkeys^49,50^ and dogs^51–53^ require a considerable amount of training before IOP can be measured without sedation, rabbits were selected as model for the current study. The rabbit is an established model for studying bleb survival and prevention of scarring following glaucoma filtration surgery,^48^ whether devices such as the EX-PRESS shunt,^54^ Molteno implant,^55^ large-lumen glaucoma drainage device,^56^ and cannula^30^ are used or not.^57^ In addition, rabbits typically exhibit aggressive subconjunctival scarring reactions, which provides an opportunity to investigate novel antifibrotics *in vivo* in an accelerated manner.^58,59^

Aqueous angiography was first combined with a tracer (fluorescein or ICG) in enucleated eyes to demonstrate segmental AHO patterns and confirm that it could simulate live AHO imaging.^60–62^ Studies in living, nonhuman primates then demonstrated that aqueous angiography could exhibit segmental, pulsatile, and dynamic patterns.^63^ Studies in humans confirmed the pulsatile and dynamic nature of AHO,^64–67^ and showed that implantation of a MIGS device (iStent; Glaukos, San Clemente, CA) affected AHO patterns.^64^ However, normal, real-time AHO has not been well characterized in rabbits, despite the importance of such models to improve surgical devices and methodologies. Altogether, these studies established aqueous angiography as a clinically relevant AHO assessment technique that can provide information pertinent to the improvement of filtration glaucoma surgeries, and set the stage for our study, the first to describe AHO pre- and postimplantation of the gel stent in a live rabbit model using aqueous angiography.

Our preimplantation findings indicated that normal AHO in the rabbit was also segmental, dynamic, and pulsatile. Description of the outflow pathway was similar to those in the aforementioned studies, with initial ICG-positive regions at the perilimbal trabecular plexus that then drained to distal areas. These results confirmed that aqueous angiography is an effective technique that can be used to refine understanding of the dynamic nature of AHO in rabbits and to evaluate potential therapies intended/designed to enhance AHO in humans.

Following ab-interno implantation of the gel stent in the rabbit, AHO changed and preferential drainage into a subconjunctival bleb was observed. Redirection of the aqueous humor from the normal, conventional pathway to the subconjunctival bleb (through the gel stent), along with the IOP lowering data, indicate that the gel stent provides an efficient, alternative pathway to restore AHO to the subconjunctival space and lower IOP, in line with clinical findings.^68^ Compared with rabbits, IOP lowering occurred with a much lower rate of subconjunctival fibrosis in human patients implanted with the gel stent, especially when an intraoperative antifibrotic was used.^23,69–73^

In the OCT images of rabbits that received the gel stent (Groups 2 [not shown] and 3), there was an increase in the number of superficial conjunctival lumens, and they were enlarged when compared to the untreated controls (Group 1). This is in line with previous studies, in which angiographically positive areas had an increased number of lumens and larger lumens than angiographically negative areas.^61–63^ The enlarged lumens were consistent with enhanced AHO through the bleb and reminiscent of lymphatic outflow lumens. In the future, this model may be used to determine the surface vasculature and visualize conjunctival lymphatics to inform on the placement of MIBS devices.

A limitation of this study is that the animal model used was nonglaucomatous rabbits aged 6–10 months (adolescent or young adults^74^) that may not fully replicate the AHO changes that occur in glaucoma models and older patients with glaucoma. Notably, in studies evaluating various pharmacologic interventions to reduce postoperative fibrosis and bleb failures after incisional glaucoma filtration surgery in rabbit models,^28–35^ the age of the rabbits used was 12–14 weeks^30^ (ie, younger than in our study), 8–12 months^34^ (ie, slightly older than in our study), or not reported.^29,31–33,35^ Data should also be interpreted with caution as the overall sample size was relatively small, as is typical of initial preclinical studies evaluating novel animal models and innovative devices.^34,35^

Although there is currently no literature to indicate that the vasculature is different between NZW and DB rabbits, other differences were reported^74–76^ and both species were initially used, which may also be a limitation. As mentioned above, the decision to switch to NZW rabbits for gel stent implantation was based on angle assessments indicating larger angles in NZW rabbits and making them more suitable for devices such as the gel stent. However, rabbits exhibit accelerated subconjunctival fibrosis, compared with humans, and caution is advised when translating results of antifibrotic studies in this model (as any model) to humans. It is also worth noting that DB rabbits were previously shown to fail glaucoma surgery sooner than NZW rabbits (as evidenced by an increase in IOP),^75^ perhaps suggesting that the NZW rabbit is a less fibrotic, more appropriate model to evaluate glaucoma filtration surgeries.

Finally, whether the gel stent-related changes in AHO adversely affect the natural outflow pathways was not evaluated and should be considered when interpreting the data.

## Conclusions

Overall, findings showed that (1) the NZW rabbit is a good model to evaluate gel stent implantation devices and bleb persistence/survival in relationship to IOP lowering, and (2) MMC SR was more effective than MMC solution in prolonging bleb survival and IOP lowering. While additional studies are needed to validate this model with other antifibrotics, the correlative results from human studies regarding the use of MMC with the gel stent and the resulting change in AHO support its use.

## Data Sharing Statement

AbbVie is committed to responsible data sharing regarding the studies we sponsor. This includes access to analysis data sets, as well as other information (eg, protocols, study reports, or analysis plans), as long as the studies are not part of an ongoing or planned regulatory submission. This includes requests for study data for unlicensed products and indications.

These study data can be requested by any qualified researchers who engage in rigorous, independent, scientific research, and will be provided following review and approval of a research proposal, Statistical Analysis Plan (SAP), and execution of a Data Sharing Agreement (DSA). Data requests can be submitted at any time after approval in the US and Europe and after acceptance of this article for publication. The data will be accessible for 12 months, with possible extensions considered. Data reported in this article may be requested by contacting AbbVie Inc.

## Authors' Contribution

Conceptualization of study: S.S.L, S.N., L.R., W.O., A.A., and M.R.R.; Statistical analysis of data: L.Y.; Data interpretation: All authors; Critical review, revisions, and approval of the article: All authors.
